# Nest inundation from sea-level rise threatens sea turtle population viability

**DOI:** 10.1098/rsos.150127

**Published:** 2015-07-22

**Authors:** David A. Pike, Elizabeth A. Roznik, Ian Bell

**Affiliations:** 1 College of Marine and Environmental Sciences, James Cook University, Townsville, Queensland 4811, Australia; 2 Department of Environment and Heritage Protection, Townsville, Queensland 4814, Australia

**Keywords:** *Chelonia mydas*, climate change, egg incubation, immersion, nesting habitat, nest flooding

## Abstract

Contemporary sea-level rise will inundate coastal habitats with seawater more frequently, disrupting the life cycles of terrestrial fauna well before permanent habitat loss occurs. Sea turtles are reliant on low-lying coastal habitats worldwide for nesting, where eggs buried in the sand remain vulnerable to inundation until hatching. We show that saltwater inundation directly lowers the viability of green turtle eggs (*Chelonia mydas*) collected from the world's largest green turtle nesting rookery at Raine Island, Australia, which is undergoing enigmatic decline. Inundation for 1 or 3 h reduced egg viability by less than 10%, whereas inundation for 6 h reduced viability by approximately 30%. All embryonic developmental stages were vulnerable to mortality from saltwater inundation. Although the hatchlings that emerged from inundated eggs displayed normal physical and behavioural traits, hypoxia during incubation could influence other aspects of the physiology or behaviour of developing embryos, such as learning or spatial orientation. Saltwater inundation can directly lower hatching success, but it does not completely explain the consistently low rates of hatchling production observed on Raine Island. More frequent nest inundation associated with sea-level rise will increase variability in sea turtle hatching success spatially and temporally, due to direct and indirect impacts of saltwater inundation on developing embryos.

## 1. Introduction

Sea-level rise is an impending threat to terrestrial fauna inhabiting coastal ecosystems [1–3]. This process will result in loss of terrestrial habitats and the creation of suboptimal habitats, which could disrupt the life histories of terrestrial species, especially those reliant on low-lying coastal or insular environments to complete their life cycles [2,4–6]. Before habitat becomes permanently lost, rising tides will inundate the terrestrial habitat more frequently [6]. Periodic, unpredictable inundation by seawater can disrupt the reproduction of coastal nesting species by directly impacting vulnerable life stages (e.g. washing away nests, drowning eggs or altricial young) and through longer term changes in habitat (e.g. substrate composition, salinity, availability of nest construction materials) [3,6–13]. Understanding how sea-level rise will impact animal populations is therefore essential for effective conservation management, but for many fauna we lack this information.

Sandy shores support special ecological features that foster high biodiversity, and these areas are at risk from the combined effects of rising sea levels and escalating anthropogenic pressures [2]. Tidal inundation can substantially alter the abiotic and geomorphological features of beaches over daily, seasonal and annual timescales [13]. Elevated sea levels, combined with tidal surges during storms, contribute to inundating low-lying coastal habitats. These effects are spatially and temporally variable, which could render some populations more vulnerable than others [14]. Sea turtles bury their eggs on sandy tropical and temperate beaches worldwide [15], where the eggs remain vulnerable to environmental conditions during the six- to eight-week incubation period. Field evidence demonstrates that inundation during incubation significantly lowers sea turtle egg viability [3,7–13,16], but we do not know how long eggs can withstand inundation and still hatch successfully. Some reports suggest that sea turtle eggs drown after only a few minutes of inundation [8], and further, that moist incubation environments can lower hatching success, especially during early stages of embryonic development [3]. This occurs because developing embryos require two-way gas exchange across the eggshell, which is impeded by submergence in water [17].

We experimentally tested how the duration of inundation by saltwater influences sea turtle egg viability. This information will increase our ability to predict how rising sea levels will impact hatching success and long-term population dynamics. Our study focuses on the largest green turtle (*Chelonia mydas*) population in the world, which nests on Raine Island in the northern Great Barrier Reef, Australia. Hatchling production is declining markedly at this site, which has been partially attributed to tidal inundation, storm overwash and heavy rainfall drowning nests [8]. From 2011 to 2015, annual hatching success of nests laid on Raine Island ranged from 12 to 36% (*n*=352 nests monitored over four nesting seasons [18]); this is substantially lower than the hatching success of other green turtle populations worldwide, which typically exceeds 80% [19,20]. Dissections of unhatched eggs in the field revealed that mortality typically occurs within the first few weeks of incubation. Our aim was therefore to test whether saltwater inundation of incubating eggs could be a primary driver of low hatching success rates at this site, which is undergoing enigmatic decline.

## 2. Material and methods

We collected green turtle eggs from Raine Island, a remote coral cay located in the northern Great Barrier Reef that supports the world's largest green turtle population (32 ha in size; 11°35′25″ S,144°2′7″ E; the closest mainland port is Cairns, 630 km away). Raine Island could lose 7–27% of its area with a mean sea-level rise of 0.18–0.79 m, as estimated by combining detailed measures of topography and elevation with Intergovernmental Panel on Climate Change predictions [6].

Eggs (*n*=262) were collected from three nesting females on 2 April 2014 and transported to Townsville for incubation. During transport from this remote site (48 h by boat, airplane and car), eggs were maintained at 4–6°C to arrest embryonic development [21]. Transporting eggs in this manner over this length of time typically does not lower their viability [21]. Eggs were individually weighed and numbered, and haphazardly split among plastic incubation trays (*n*=14;285×210×70 mm). Incubation trays contained a single layer of 13–20 eggs, separated by moist vermiculite (1 : 1 vermiculite to rainwater mass). Trays were covered with plastic wrap to prevent evaporative water loss, and split between two incubators (180×60×60 cm, each containing seven shelves). Thermochron iButton dataloggers (factory calibrated, accurate to ±0.5°C) placed on top of each egg tray were used to confirm that incubation temperatures maintained 29.5 ± 0.5°C throughout both incubators during incubation.

We randomly allocated trays to receive saltwater inundation treatments at different points during embryonic development: just laid, one-third developed, one-half developed or two-thirds developed, or full-term controls which were not inundated (table 1). Within each tray, we randomly allocated each egg to one of four treatments: no inundation (control), or saltwater inundation for 1, 3 or 6 h (table 1). These inundation times broadly approximate potential exposure of real nests to seawater during an unusually high tide, with the lowest lying nests being inundated for a maximum of 6 h and more elevated nests having progressively less exposure. Each treatment egg was inundated only once during development. Before inundating trays, we carefully moved the control eggs to a new incubation tray. We poured saltwater (28 ppt, specific gravity =1.025; mixed from rainwater and ReefSalt (Seachem Laboratories Inc, Madison, GA, USA)) over the remaining eggs until they were completely covered. After inundation, eggs were transferred to the new incubation tray with the control eggs and left undisturbed until hatching.

**Table 1.** Experimental design, detailing the numbers of eggs used in each inundation treatment. We observed high egg mortality that was unrelated to inundation, which was first detectable on day 12 of incubation. Because of this, all eggs from the day 2 treatment appeared viable at inundation, and the full-term day 50 treatment acts as a control without any inundation. Numbers in parentheses indicate eggs that appeared viable at the time of saltwater immersion. The dataset is available in the electronic supplementary material.

	day of incubation and embryonic stage					
inundation treatment	day 2 just laid	day 12 one-third developed (known viable)	day 25 one-half developed (known viable)	day 37 two-thirds developed (known viable)	Day 50 full-term	Total N (known viable)
no inundation	13	13 (6)	13 (6)	13 (6)	54	106 (18)
saltwater—1 h	13	13 (9)	13 (5)	13 (5)	—	52 (19)
saltwater—3 h	13	13 (8)	13 (4)	13 (8)	—	52 (20)
saltwater—6 h	13	13 (10)	13 (2)	13 (5)	—	52 (17)

We monitored the fate of each egg (hatched or died) and euthanized hatchlings using 0.1 *ml* of Valabarb. During the first 12 days of incubation, we observed high rates of egg mortality (shrivelling, discoloration) that was unrelated to our inundation treatments, which also occurs in nests on Raine Island [8]. To account for this, we scored whether the remaining eggs (one-third developed, one-half developed, two-thirds developed and full-term controls) appeared viable immediately before inundation. This allowed us to control our analysis for the effects of high egg mortality that were unrelated to saltwater inundation. Eggs scored as viable were ivory in colour and turgid, whereas those deemed unviable were discoloured and often had shrivelled to less than half their initial volume. All eggs inundated just after being laid appeared viable at that time (but could have been unviable and we could not detect this so early during development), and eggs in the full-term control treatment were not disturbed until hatching (table 1).

We used a generalized linear model in program R (v. 3.1.2) [22] to investigate potential drivers of hatching success, including the effects of timing of inundation during embryonic development, inundation treatment, initial egg mass and apparent egg viability prior to inundation. To avoid overfitting the model to our small dataset, we did not include interactions.

## 3. Results

**Figure 1.**
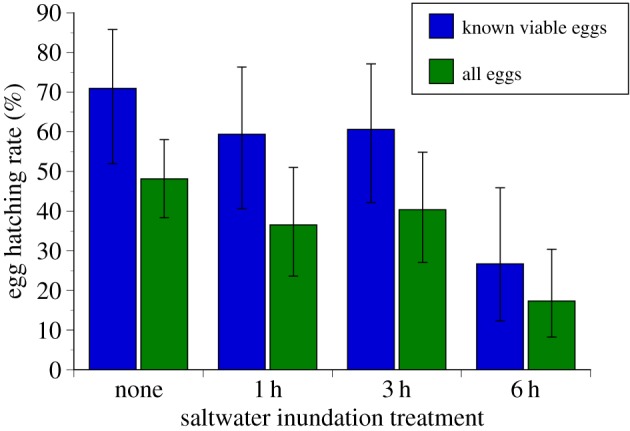
Hatching success of green turtle (*Chelonia mydas*) eggs immersed in saltwater, calculated using all eggs (*n*=262) and using only eggs that appeared viable at saltwater immersion (*n*=126). Error bars represent 95% confidence limits.

Green turtle eggs inundated by saltwater for 6 h had significantly lower hatching success than control eggs, by approximately 30% (table 2 and figure 1). Relatively short inundation—1 or 3 h—reduced egg viability by less than 10%, which was not significantly different from the hatching success of control eggs (table 2 and figure 1). We observed high egg mortality in the control eggs overall (45%), and our analysis revealed that whether eggs appeared viable immediately prior to inundation was related to hatching success (table 2). The estimates of hatching success that include all of the eggs are significantly lower than those including only those eggs scored as viable prior to inundation (table 2 and figure 1). In terms of the timing of inundation, the only significant result was that hatching success was lower immediately after oviposition (when all eggs appeared viable, but we may not have been able to distinguish unviable eggs) when compared with all other embryonic stages (when we could successfully differentiate viable and unviable eggs). This result is probably spurious, and due to the inclusion of non-viable eggs in the estimates of hatching success for the eggs exposed at oviposition. Saltwater immersion had no effect on incubation duration; hatchlings emerged within 51–60 days, and displayed typical physical and behavioural traits.

**Table 2.** Results of a generalized linear model examining effects of inundation time (1, 3, 6 h or not inundated), day of exposure (day 2, 12, 25, 37 or never inundated), initial egg mass and viability prior to inundation on hatching success of green turtle eggs. Shown are estimates for the coefficients included in the model, their s.e., *t*-values and *p*-values. Significant values appear in italics.

coefficient	estimate	s.e.	*t*-value	*p*-value
intercept	0.2624	0.4040	0.6500	0.5166
initial egg mass	−0.0035	0.0089	−0.3930	0.6948
egg viability	0.7913	0.0454	17.4280	<*0.0001*
inundation time—1 h	−0.1133	0.0629	−1.8000	0.0731
inundation time—3 h	−0.0891	0.0628	−1.4180	0.1573
inundation time—6 h	−0.2732	0.0628	−4.3540	<*0.0001*
day of exposure—2	−0.5269	0.0764	−6.8980	<*0.0001*
day of exposure—12	−0.0252	0.0734	−0.3430	0.7316
day of exposure—25	0.0439	0.0736	0.5970	0.5510
day of exposure—37	0.0540	0.0732	0.7370	0.4618

## 4. Discussion

Female turtles select nest sites that favour high egg viability; nests that are located farther inland and are more elevated are less vulnerable to inundation, but when nests are located too far from the ocean the emerging hatchlings have difficulty migrating to the ocean [7]. Habitat availability therefore constrains nest placement, and this can render nests in even the most elevated positions vulnerable to inundation [3,12]. Short periods of saltwater inundation, such as those associated with high tides during severe storms, substantially lower green turtle egg viability (figure 1). Saltwater inundation is not universally lethal, however, and some incubating embryos survived and hatched after 6 h of inundation by saltwater.

Raine Island supports the world's largest green turtle rookery, but nesting success and hatchling production have been declining for nearly two decades [8]. Nests laid on Raine Island consistently have high egg mortality during early development, even when incubation conditions are ideal [8]. Consistent with field data, we observed high rates of unexplained egg mortality within two weeks of oviposition, which also affected our full-term control treatment. This unexplained mortality influenced our comparison of hatching success among the times of inundation with respect to developmental stage. Hatching success was significantly lower in the treatment inundated immediately after oviposition (when all eggs appeared viable) than all other incubation treatments (in which we could clearly distinguish viable from unviable eggs; table 1). This result probably reflects the unavoidable inclusion of unviable eggs into our experiment and does not indicate that eggs are more vulnerable to inundation early in development. Therefore, it appears as though all developmental stages we tested are equally vulnerable to the saltwater inundation treatments we used, although further testing is warranted given the high rate of unviable eggs included in our study.

Inundation probably contributes to low hatching success rates in the field at Raine Island [8], but the high rates of unviable eggs (approx. 45%) strongly imply that there are other factors causing embryonic development to cease early during incubation. These could include a wide range of interrelated factors, ranging from the health of mother turtles, which can affect hatching success [23], to contaminants transferred from mothers to eggs, which can disrupt development [24], and/or high levels of microbes present in the soil, which can reduce oxygen availability for embryos [25]. It is also possible that repeated inundation of field nests (as opposed to a single event, which we tested) could further reduce hatching success, and this warrants further testing. Overall, however, the enigmatic decline in emerging hatchlings on Raine Island remains unexplained and probably is not primarily a result of nest inundation by seawater.

Short periods of hypoxia during incubation caused by saltwater immersion directly reduces the viability of sea turtle embryos, but we do not yet know whether this has other sublethal effects on neurological function that could influence behaviour, learning or spatial orientation in hatchlings [26]. Rising sea levels and storms will have profound effects on coastal habitats by inundating eggs, reducing nesting areas by altering beach geomorphology, depositing new sand, or depositing debris that can act as barriers for nesting females or emerging hatchlings [13]. Rising sea levels will also be accompanied by higher wave run-up during storms [6], which will likely increase mortality of sea turtle eggs laid in low-lying areas by drowning a portion of the incubating eggs.

## Supplementary Material

Dataset

## Data accessibility

The dataset is available in the electronic supplementary material.

## References

[1] Fish MR *et al.* 2005 Predicting the impact of sea-level rise on Caribbean sea turtle nesting habitat. *Conserv. Biol.* 19, 482–491. (doi:10.1111/j.1523-1739.2005.00146.x)

[2] Schlacher TA *et al* 2007 Sandy beaches at the brink. *Divers. Distrib.* 13, 556–560. (doi:10.1111/j.1472-4642.2007.00363.x)

[3] Patino-Martinez J, March A, Quiñones L, Hawkes LA 2014 The potential future influence of sea level rise on leatherback turtle nests. *J. Exp. Mar. Biol. Ecol.* 461, 116–123. (doi:10.1016/j.jembe.2014.07.021)

[4] Ranasinghe R, Stive MJF 2009 Rising seas and retreating coastlines. *Clim. Change* 97, 465–468. (doi:10.1007/s10584-009-9593-3)

[5] LaFever DH, Lopez RR, Feagin RA, Silvy NJ 2007 Predicting the impacts of future sea-level rise on an endangered lagomorph. *Environ. Manage.* 40, 430–437. (doi:10.1007/s00267-006-0204-z) 17557173 10.1007/s00267-006-0204-z

[6] Fuentes MMPB, Limpus CJ, Hamann M, Dawson J 2010 Potential impacts of projected sea-level rise on sea turtle rookeries. *Aquat. Conserv.* 20, 132–139. (doi:10.1002/aqc.1088)

[7] Wood DW, Bjorndal KA 2000 Relation of temperature, moisture, salinity, and slope to nest site selection in loggerhead sea turtles. *Copeia* 2000, 119–128. (doi:10.1643/0045-8511(2000)2000[0119:ROTMSA]2.0.CO;2)

[8] Limpus CJ, Miller JD, Parmenter CJ, Limpus DJ 2003 The green turtle, *Chelonia mydas*, population of Raine Island and the Northern Great Barrier Reef: 1843–2001. *Mem. Queensl. Mus.* 49, 349–440.

[9] Pike DA, Stiner JC 2007 Sea turtle species vary in their susceptibility to tropical cyclones. *Oecologia* 153, 471–478. (doi:10.1007/s00442-007-0732-0) 17479295 10.1007/s00442-007-0732-0

[10] Pike DA, Stiner JC 2007 Fluctuating reproductive output and environmental stochasticity: do years with more reproducing females result in more offspring? *Can. J. Zool.* 85, 737–742. (doi:10.1139/Z07-055)

[11] Van Houtan KS, Bass OL 2007 Stormy oceans are associated with declines in sea turtle hatching. *Curr. Biol.* 17, 590–591. (doi:10.1016/j.cub.2007.06.021) 10.1016/j.cub.2007.06.021 17686427

[12] Caut S, Guirlet E, Girondot M 2010 Effect of tidal overwash on the embryonic development of leatherback turtles in French Guiana. *Mar. Environ. Res.* 69, 254–261. (doi:10.1016/j.marenvres.2009.11.004) 19969341 10.1016/j.marenvres.2009.11.004

[13] Behera SK, Mohanta RK, Kar C, Mishra SS 2014 Impacts of the super cyclone Philine on sea turtle nesting habitats at the Rushikulya Rookery, Ganjam Coast, India. *Poult. Fisheries Wildl. Sci.* 2, 114.

[14] Dewald JR, Pike DA 2014 Geographical variation in hurricane impacts among sea turtle populations. *J. Biogeogr.* 41, 307–316. (doi:10.1111/jbi.12197)

[15] Pike DA 2013 Climate influences the global distribution of sea turtle nesting. *Glob. Ecol. Biogeogr.* 22, 555–566. (doi:10.1111/geb.12025)

[16] Fuentes MMPB, Bateman BL, Hamann M 2011 Relationship between tropical cyclones and the distribution of sea turtle nesting grounds. *J. Biogeogr.* 38, 1886–1896. (doi:10.1111/j.1365-2699.2011.02541.x)

[17] Ackerman RA 1997 The nest environment and the embryonic development of sea turtles. In *The biology of sea turtles* (eds PL Lutz, JA Musick), pp. 83–86 Boca Raton, FL: CRC Press.

[18] Dunstan A, Norris B, Sievers W 2015 *Raine Island turtle recovery project report 2014–2015 season*, p. 46 Brisbane, Australia: Department of Environment and Heritage Protection.

[19] Pike DA 2008 Natural beaches confer fitness benefits to nesting marine turtles. *Biol. Lett.* 4, 704–706. (doi:10.1098/rsbl.2008.0359) 18765355 10.1098/rsbl.2008.0359 PMC2614151

[20] Pike DA 2009 Natural beaches produce more hatchling marine turtles than developed beaches, despite regional differences in hatching success. *Biol. Lett.* 5, 268–269. (doi:10.1098/rsbl.2008.0693)

[21] Harry JL, Limpus CJ 1989 Low-temperature protection of marine turtle eggs during long-distance relocation. *Aust. Wildl. Res.* 16, 317–320. (doi:10.1071/WR9890317)

[22] R Core Team. 2014 *R: a language and environment for statistical computing*. Vienna, Austria: R Foundation for Statistical Computing. See http://www.R-project.org/.

[23] Perrault JR, Miller DL, Eads E, Johnson C, Merrill A, Thompson LJ, Wyneken J 2012 Maternal health status correlates with nest success of leatherback sea turtles (*Dermochelys coriacea*) from Florida. *PLoS ONE* 7, e31841 (doi:10.1371/journal.pone.0031841). 22359635 10.1371/journal.pone.0031841 PMC3281022

[24] van de Merwe JP, Hodge M, Whittier JM, Ibrahim K, Lee SY 2010 Persistent organic pollutants in the green sea turtle *Chelonia mydas*: nesting population variation, maternal transfer, and effects on development. *Mar. Ecol. Prog. Ser.* 403, 269–278. (doi:10.3354/meps08462)

[25] Sarmiento-Ramírez JM, Abella-Pérez E, Phillott AD, Sim J, van West P, Martín MP, Marco A, Diéguez-Uribeondo J 2014 Global distribution of two fungal pathogens threatening endangered sea turtles. *PLoS ONE* 9, e85853 (doi:10.1371/journal.pone.0085853). 24465748 10.1371/journal.pone.0085853 PMC3897526

[26] Singer D 1999 Neonatal tolerance to hypoxia: a comparative-physiological approach. *Comp. Biochem. Physiol. A: Mol. Integr. Physiol.* 123, 221–234. (doi:10.1016/S1095-6433(99)00057-4) 10501017 10.1016/s1095-6433(99)00057-4

